# HIF-1α contributes to Ang II-induced inflammatory cytokine production in podocytes

**DOI:** 10.1186/s40360-019-0340-8

**Published:** 2019-10-17

**Authors:** Hao Huang, Yanqin Fan, Zhao Gao, Wei Wang, Ning Shao, Lu Zhang, Yingjie Yang, Weifang Zhu, Zhaowei Chen, Jijia Hu, Guohua Ding

**Affiliations:** 1 Division of Nephrology, Renmin Hospital of Wuhan University, Wuhan, 430060 Hubei China; 2 Division of Rehabilitation, Tianmen First People’s Hospital, Tianmen, Hubei China

**Keywords:** HIF-1α, Podocyte, Angiotensin II, Inflammatory cytokine

## Abstract

**Background:**

Studies have indicated that changed expression of hypoxia-inducible factor-1α (HIF-1α) in epithelial cells from the kidney could affect the renal function in chronic kidney disease (CKD). As Angiotensin II (Ang II) is a critical active effector in the renin-angiotensin system (RAS) and was proved to be closely related to the inflammatory injury. Meanwhile, researchers found that Ang II could alter the expression of HIF-1α in the kidney. However, whether HIF-1α is involved in mediating Ang II-induced inflammatory injury in podocytes is not clear.

**Methods:**

Ang II perfusion animal model were established to assess the potential role of HIF-1α in renal injury in vivo. Ang II stimulated podocytes to observe the corresponding between HIF-1α and inflammatory factors in vitro*.*

**Results:**

The expression of inflammatory cytokines such as MCP-1 and TNF-α was increased in the glomeruli from rats treated with Ang II infusion compared with control rats. Increased HIF-1α expression in the glomeruli was also observed in Ang II-infused rats. In vitro, Ang II upregulated the expression of HIF-1α in podocytes. Furthermore, knockdown of HIF-1α by siRNA decreased the expression of MCP-1 and TNF-α. Moreover, HIF-1α siRNA significantly diminished the Ang II-induced overexpression of HIF-1α.

**Conclusion:**

Collectively, our results suggest that HIF-1α participates in the inflammatory response process caused by Ang II and that downregulation of HIF-1α may be able to partially protect or reverse inflammatory injury in podocytes.

## Background

Podocytes are terminally differentiated cells that play a crucial role in maintaining the integrity of the glomerular filtration barrier. Accumulating evidence has demonstrated that podocyte injury is a key determinant of proteinuric kidney disease and is associated with the progression of diabetic kidney disease (DKD) [1].

Activation of the renin-angiotensin system (RAS) promotes the occurrence and development of chronic kidney diseases (CKD). As an important active effector in the RAS, angiotensin II (Ang II) is a well-known risk factor for the initiation and progression of renal disease. Our previous studies demonstrated that Ang II induces podocyte apoptosis, cytoskeletal rearrangement and cholesterol accumulation, which could contribute to the onset of proteinuria and the progression of CKD [2–4]. However, further clarification of the exact molecular mechanism of Ang II-induced podocyte injury remains necessary.

Hypoxia-inducible factor (HIF) is a transcription factor that regulates the adaptive response to hypoxia. It is composed of an oxygen-sensitive α subunit and a stable β subunit [5]. HIF-1α is expressed in all cells and has been detected in the renal tubular epithelium (RTE) [6]. A prior study showed that renal injury was accelerated by HIF-1α deficiency in an STZ-induced diabetic animal model [7]. HIF-1α induction prevented excessive accumulation of lipids in alcoholic fatty liver in an animal model [8]. However, the specific mechanism of HIF-1α in podocyte injury is not clear. This study aimed to assess the role of HIF-1α in Ang II-induced podocyte injury. It may offer potential clinical applications for CKD.

## Methods

### Animals

Twenty male SPF Wistar rats weighing between 140 and 160 g were provided by the Hubei Research Center of Experimental Animals (Hubei, China). The animals were housed under an artificial light cycle at a controlled room temperature (23 ± 2 °C) and humidity (55 ± 5%). After implantation of an osmotic mini-pump (Alzet model 2002 or 2004, CA), rats were randomly assigned to the control group subjected to normal saline infusion or the experimental group subjected to Ang II (Sigma Aldrich, USA) infusion (400 ng/kg/min) for 56 days. The rats in both groups had free access to tap water and standard rat chow. Blood pressure changes (0 weeks, 2 weeks, 4 weeks, 8 weeks) were measured and recorded at different time points using a rat tail non-invasive blood pressure meter. The animals were sacrificed on day 56. At the end of the experiments, mice were sacrificed by euthanasia using isoflurane inhalation and kidneys was collected. Kidneys were infused with vanadate (a phosphatase inhibitor) before being harvested and stored at − 80 °C for biochemical and renal pathological analysis.

### Cell culture

Conditionally immortalized human podocytes were kindly provided by Dr. Moin A. Saleem (Academic Renal Unit, Southmead Hospital, Bristol, UK). These cells were incubated at 33 °C in RPMI 1640 medium (HyClone, USA) containing 10% fetal bovine serum (FBS) (Gibco, USA), 100 U/mL penicillin G, 100 μg/mL streptomycin, and 1× insulin, transferrin, and selenium (ITS) (Invitrogen, USA) in the presence of 5% CO_2_. When the podocytes had proliferated to approximately 80% confluence, they were transferred to a 37 °C incubator with ITS-free medium for 7–14 days to induce differentiation. Differentiated podocytes were serum starved in RPMI 1640 medium without FBS for 24 h before all experiments. The cells were then treated with 10^− 8^–10^− 5^ M Ang II (Sigma-Aldrich, USA).

### Western blotting

Total protein was extracted from tissues and cells and lysed in RIPA buffer (Beyotime, China) mixed with protease/phosphatase inhibitors (Sigma-Aldrich, USA). Samples were collected via centrifugation at 13,000 g and 4 °C for 5 min. The supernatants were boiled at 100 °C for 5 min in loading buffer. A BCA protein assay (Thermo Scientific, USA) was used to measure protein concentrations. Equal amounts of protein were separated using 10% sodium dodecyl sulfate-polyacrylamide gel electrophoresis (SDS-PAGE) and then transferred to polyvinylidene difluoride (PVDF) membranes (Millipore, USA). The membranes were incubated overnight at 4 °C with primary antibody (HIF-1α mouse monoclonal antibody, 1:2000, Novus; TNF alpha rabbit polyclonal antibody, 1:1000, Abcam; MCP-1 mouse monoclonal antibody, 1:1000, Novus; beta tubulin rabbit polyclonal antibody, 1:1000, Proteintech; GAPDH rabbit polyclonal antibody, 1:1000, Proteintech; beta actin rabbit polyclonal antibody, 1:1000, Proteintech). An Alexa Fluor 680/790-labeled goat anti-rabbit/goat anti-mouse IgG antibody (1:25,000, LI-COR Biosciences, USA) was used as the secondary antibody. An Odyssey infrared imaging system (LI-COR Biotechnology, USA) was employed to visualize the blots.

### Cell transfection

HIF-1α small interfering RNA (siRNA) transfection was performed to silence gene expression in accordance with the HiPerFect Transfection Reagent Handbook (Qiagen, Germany). Briefly, 2 × 10^5^ cells were seeded in a six-well plate and transfected with medium containing 15 μl of HiPerFect transfection reagent and 10 nM HIF-1α siRNA or scrambled siRNA (as a negative control, SNC) under normal growth conditions for 48 h.

### Statistical analyses

All experiments were performed at least three times, with similar results obtained among replicate experiments. All values are presented as means with SDs and were analyzed using SPSS 17.0. Differences were considered to be statistically significant if *p* < 0.05.

## Results

### Effects of Ang II infusion on renal injury

**Fig. 1:**
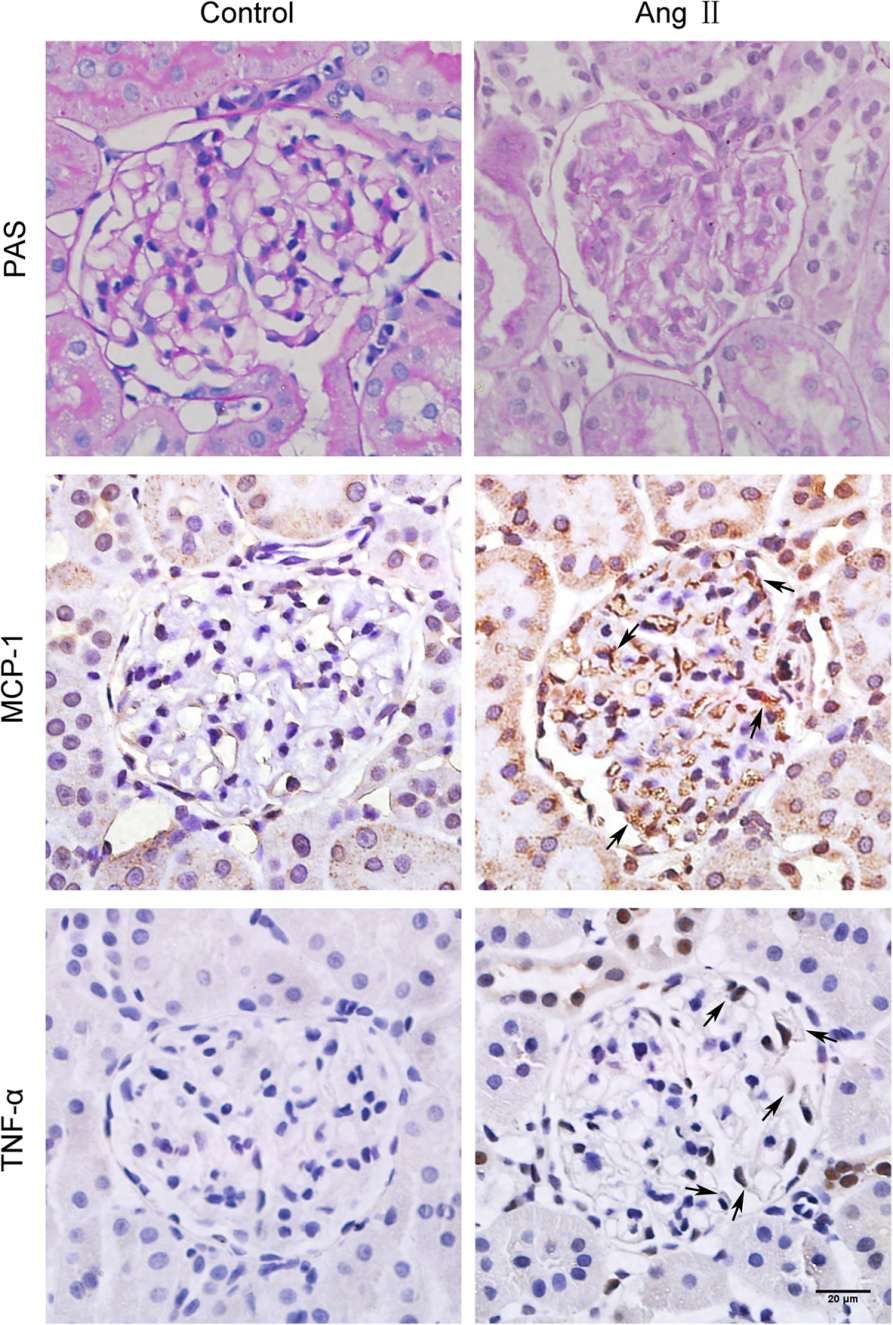
Glomerular pathological changes in different groups. Light microscopy evaluation of glomerular pathological changes with periodic acid-Schiff staining. Representative images of immunochemical staining to detect the expression of MCP-1 and TNF-α in kidneys in different groups (original magnification, × 400). Arrows pointing to the positive area of immunochemical staining. Ang II, Ang II-infused group; Control, normal saline-infused group. PAS: periodic acid-Schiff

To investigate the effects of Ang II in glomerular and podocyte injury, we established a rat model of Ang II infusion. The blood pressure increased gradually in the Ang II infusion group and the difference was statistically significant at the end of the experiment compared with the normal control group (Additional file 1). After a 8-week infusion of Ang II, Ang II-infused rats showed proliferation of glomerular mesangial cells and atrophy of renal tubular epithelial cells. To investigate the functions of inflammatory factors in renal injury, kidneys were stained for MCP-1 and TNF-α via immunohistochemistry for further analysis. Light microscopy revealed that MCP-1 and TNF-α expression in the kidneys was increased in Ang II-infused rats compared with control rats (Fig. 1).

### Effects of Ang II on HIF-1α expression in Ang II-infused rats

**Fig. 2:**
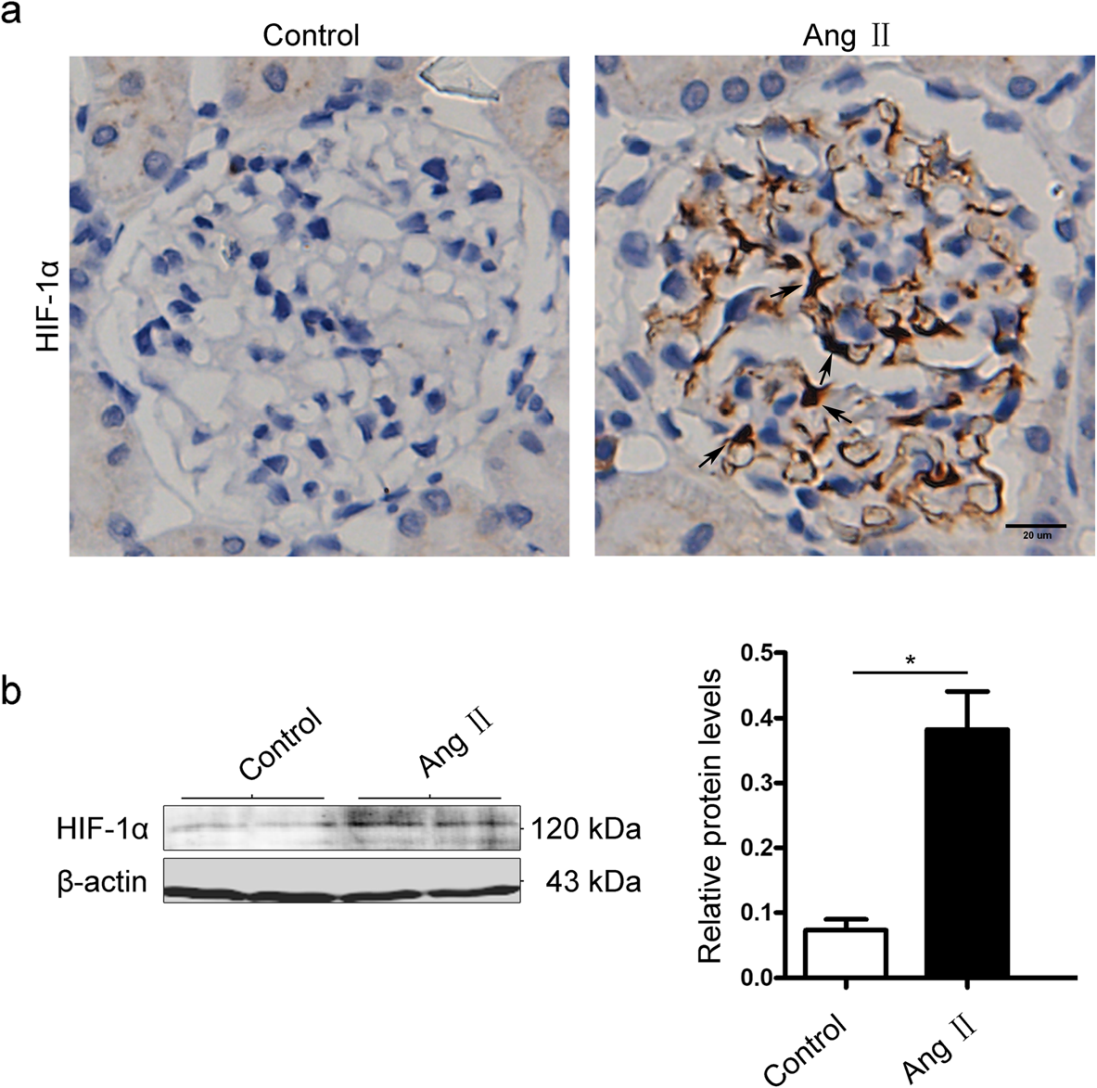
Ang II stimulation enhanced HIF-1α expression in vivo. **a** Representative images of immunochemical staining to detect HIF-1α expression in kidneys in different groups (original magnification, × 400). Arrows pointing to the positive area of immunochemical staining**. b** Representative Western blots of HIF-1α expression in different groups. Ang II, Ang II-infused group; Control, normal saline-infused group. β-actin was used as an equal loading marker. *p < 0.05 compared with the normal group at the same time point

We next evaluated the effects of Ang II on HIF-1α expression using immunohistochemistry. Almost no HIF-1α expression was detected in the control group, whereas upregulation of HIF-1α was observed in Ang II-infused rats (Fig. 2a). We then verified the expression changes of HIF-1 via Western blotting. Lower HIF-1α expression was observed in saline-treated control rats than in Ang II-infused rats. These results were the same as those observed using immunohistochemistry (Fig. 2b). Therefore, it was hypothesized whether the up-regulation of HIF-1α expression induced by Ang II was associated with the increased expression of inflammatory cytokines.

### Effects of Ang II on HIF-1α expression in cultured podocytes

**Fig. 3:**
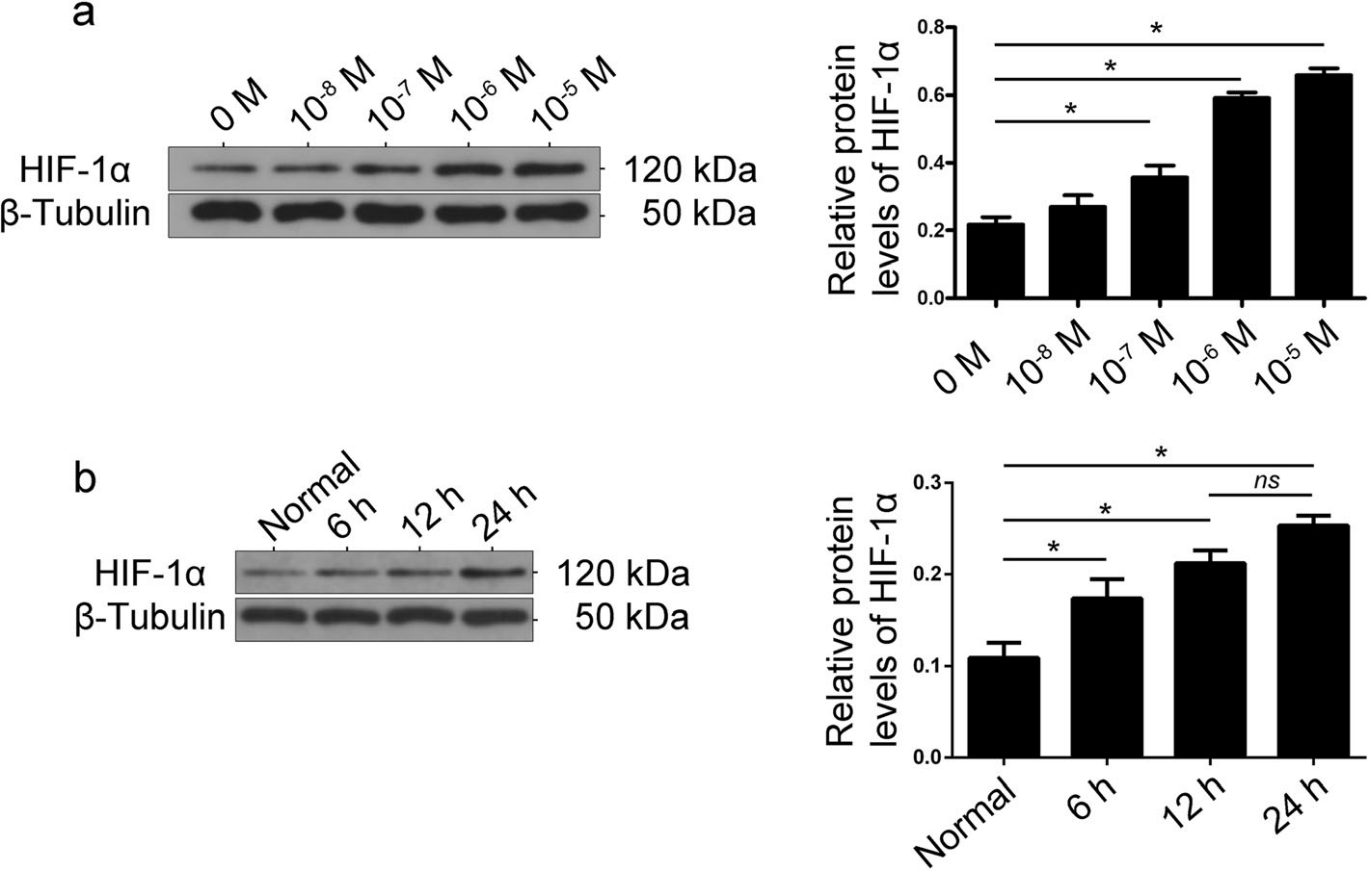
**a** Effect of Ang II on the protein expression of HIF-1α in podocytes. Podocytes were treated with various concentrations of Ang II (10^-8^–10^-5^ M) for 24 h. The bands show changes in the relative protein levels of HIF-1α, demonstrating that Ang II enhanced HIF-1α protein levels. β-Tubulin was used as an equal loading marker. (*p < 0.05 in t-test, *n* = 3). **b** The protein expression of HIF-1α in podocytes under Ang II (10^-7^ M) stimulation in different time-points. β-Tubulin was used as an equal loading marker. (*p < 0.05 in t-test, *n* = 3; *ns,* not significant)

To confirm the effect of Ang II on HIF-1α expression in vitro, cultured podocytes were treated with Ang II (10^-8^–10^-5^ M) for 24 h. As shown in Fig. 3a, the expression of HIF-1α was significantly increased in podocytes after Ang II stimulation. At the same time, up-regulation of HIF-1α expression was detected in different Ang II stimulation duration (Fig. 3b), indicating that Ang II increased HIF-1α expression in podocytes.

### Effects of HIF-1α on inflammatory cytokine production

**Fig. 4:**
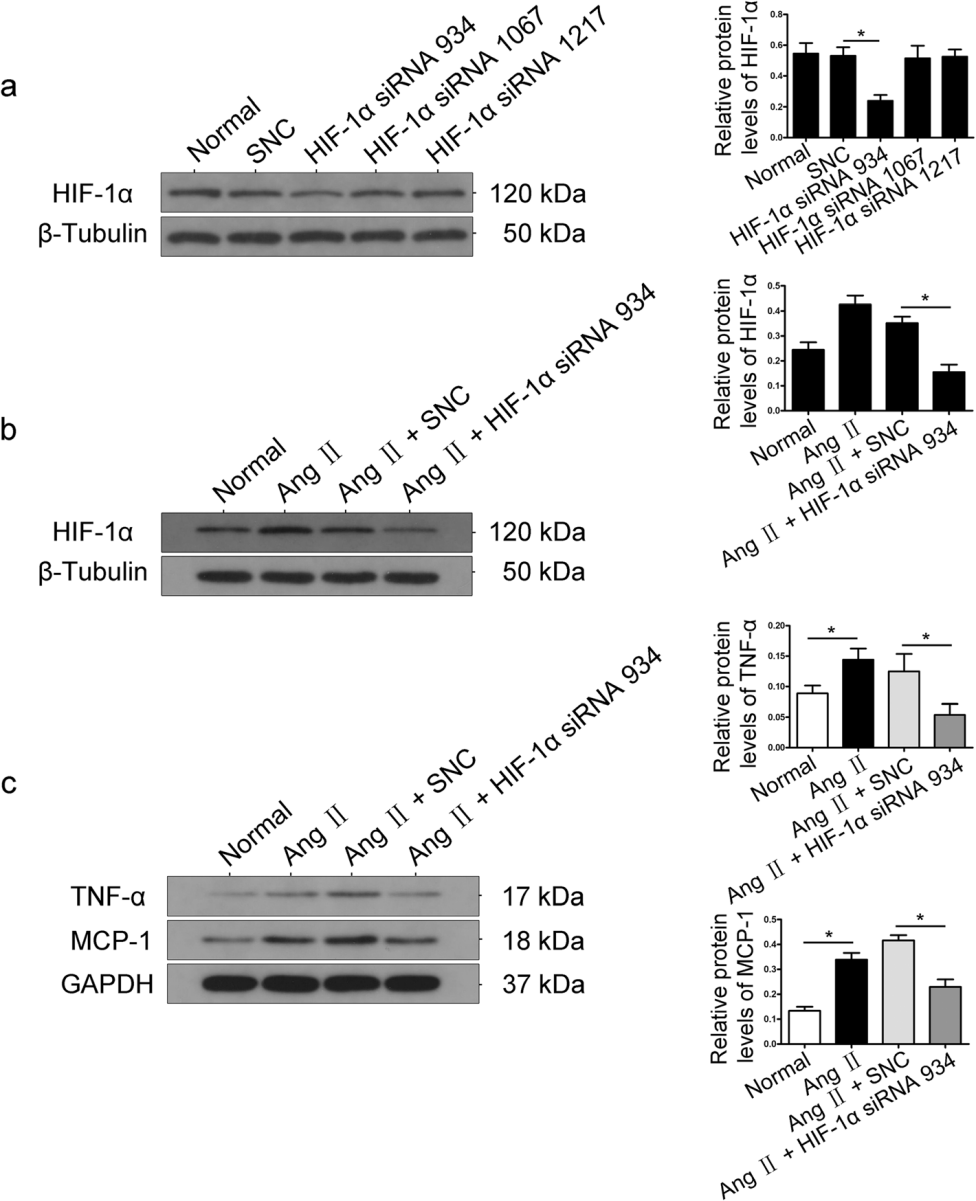
Knockdown of HIF-1α attenuated Ang II-induced inflammatory cytokine production in podocytes. **a** Podocytes were transfected with siRNA 934, 1067 or 1217 to knock down HIF-1α expression. Untreated and scramble siRNA transfected podocytes (scramble negative control, SNC) were designated as the normal group. β-Tubulin was used as an equal loading marker. (*p < 0.05 in one-way ANOVA, *n* = 3). **b** Podocytes were transfected with siRNA 934 and then stimulated with 10^-7^ M Ang II for 12 h. β-Tubulin was used as an equal loading marker. (*p < 0.05 in one-way ANOVA, *n* = 3). **c** Podocytes were transfected with siRNA 934 and then stimulated with 10^-7^ M Ang II for 12 h. Representative Western blots of MCP-1 and TNF-α expression in the different groups. β-Tubulin was used as an equal loading marker. (*p < 0.05 in one-way ANOVA, *n* = 3)

To further confirm the potential role of HIF-1α in Ang II induced inflammatory injury in podocytes, siRNAs 934, 1067 and 1217 were transfected into cultured podocytes to knock down the expression of HIF-1α. As shown in Fig. 4a, transfection with siRNA 1067 or siRNA 1217 did not affect HIF-1α expression relative to that observed in control cells. siRNA 934 transfection markedly reduced HIF-1α expression by 60% relative to that observed in control cells. Therefore, we chose siRNA 934 to knock down the expression of HIF-1α. Podocytes were transfected with siRNA 934 and then stimulated with 10^− 7^ M Ang II for 12 h. HIF-1α siRNA significantly diminished Ang II–induced overexpression of HIF-1α (Fig. 4b). Interestingly, the expression of MCP-1 and TNF-α was enhanced in Ang II–induced podocytes, whereas this effect was diminished in siRNA-transfected cells treated with Ang II (Fig. 4c), which provides the evidence for a link between HIF-1α and inflammatory injury in podocytes under Ang II stimulation.

## Discussion

A multitude of clinical and experimental studies have confirmed that Ang II plays an important role in CKD progression and podocyte injury [9, 10]. Our previous studies showed that Ang II infusion induced proteinuria and hypertension in rats and subsequently led to apoptosis of podocytes. The observed pathological manifestations of the kidneys included mesangial hyperplasia, glomerular sclerosis and renal tubule atrophy [2–4]. In the present study, we successfully induced renal injury in rats using an Ang II infusion model. We also found increased expression of inflammatory factors, such as MCP-1 and TNF-α, in the glomeruli from Ang II-infused rats. These results suggested that inflammation may contribute to Ang II-induced renal injury.

HIF-1 mainly regulates hypoxic reaction proteins, including angiogenic factors, glycolytic enzymes and cell survival proteins, a certain degree of up-regulation of HIF-1α expression can be used to alleviate anemia in CKD. Many recent studies have found that HIF-1 is associated with inflammation in tumors and have recognized HIF-1 as an important cancer drug target. One report indicated that in pancreatic ductal adenocarcinoma (PDAC), HIF-1α promoted the secretion of MCP-1, enhanced monocyte and macrophage recruitment, and strengthened inflammation and fibrosis [11]. In addition, Kihira et al. demonstrated that knockdown of HIF-1α expression reduced the mRNA expression of MCP-1 and TNF-α in epididymal visceral adipose tissue induced by a high-fat diet [12]. Thus, the effects of HIF-1α on inflammatory factors are controversial. Conde et al. demonstrated that interference with HIF-1α expression increased the expression of IL-1β, TNF-α and MCP-1 [13]. In our studies, we observed that Ang II induces upregulation of HIF-1α in vivo and in vitro. An increasing number of reports support the notion that HIF-1α contributes to renal injury and podocyte injury [14–16].

To further investigate the effect of HIF-1α in Ang II-induced inflammatory stress disorders podocyte injury, HIF-1α siRNA was used to knock down HIF-1α expression. In this study, we found that Ang II-induced inflammatory cytokines were reduced by inhibition of HIF-1α. Therefore, our hypothesis was verified that Ang II-induced podocyte inflammatory injury might be related to the overexpression of HIF-1α. Previous studies have found that silencing of HIF-1α could reduce renal injury [17] and chronic renal ischemia induced by Ang II in rats [18]. However, the role of HIF-1α in renal injury remains controversial. Certain studies have reported that HIF-1α deficiency affected the survival of early podocytes and subsequently resulted in glomerular injury [8]. In a word, further study of the regulatory mechanism of HIF-1α in Ang II-induced podocyte injury remains necessary.

## Conclusions

The present study shows that Ang II induced overexpression of HIF-1α may be related to the increased of inflammatory factors in podocytes. Therefore, HIF-1α is expected to be a new target for the diagnosis and treatment of CKD. However, a lot of research is needed to clarify the mechanism of HIF-1α in the occurrence and progress of CKD.

## Supplementary information

**Additional file 1.** Changes in blood pressure in different groups of rats.

## Data Availability

The datasets used and/or analyzed during the current study are available from the corresponding author on reasonable request.

## Abbreviations

Ang II: Angiotensin II
CKD: Chronic kidney diseases
DKD: Diabetic kidney disease
HIF: Hypoxia-inducible factor
HIF-1α: Hypoxia-inducible factor-1α
ITS: Insulin, transferrin, and selenium
PDAC: Pancreatic ductal adenocarcinoma
PVDF: Polyvinylidene difluoride
RAS: Renin-angiotensin system
RTE: Renal tubular epithelium
SDS-PAGE: Sodium dodecyl sulfate-polyacrylamide gel electrophoresis
siRNA: Small interfering RNA
