# Supplementary figures and images for: HIF-1α contributes to Ang II-induced inflammatory cytokine production in podocytes
Source: BMC Pharmacol Toxicol. 2019 Oct 17;20:59. doi: 10.1186/s40360-019-0340-8 (PMC6796393; doi:10.1186/s40360-019-0340-8)

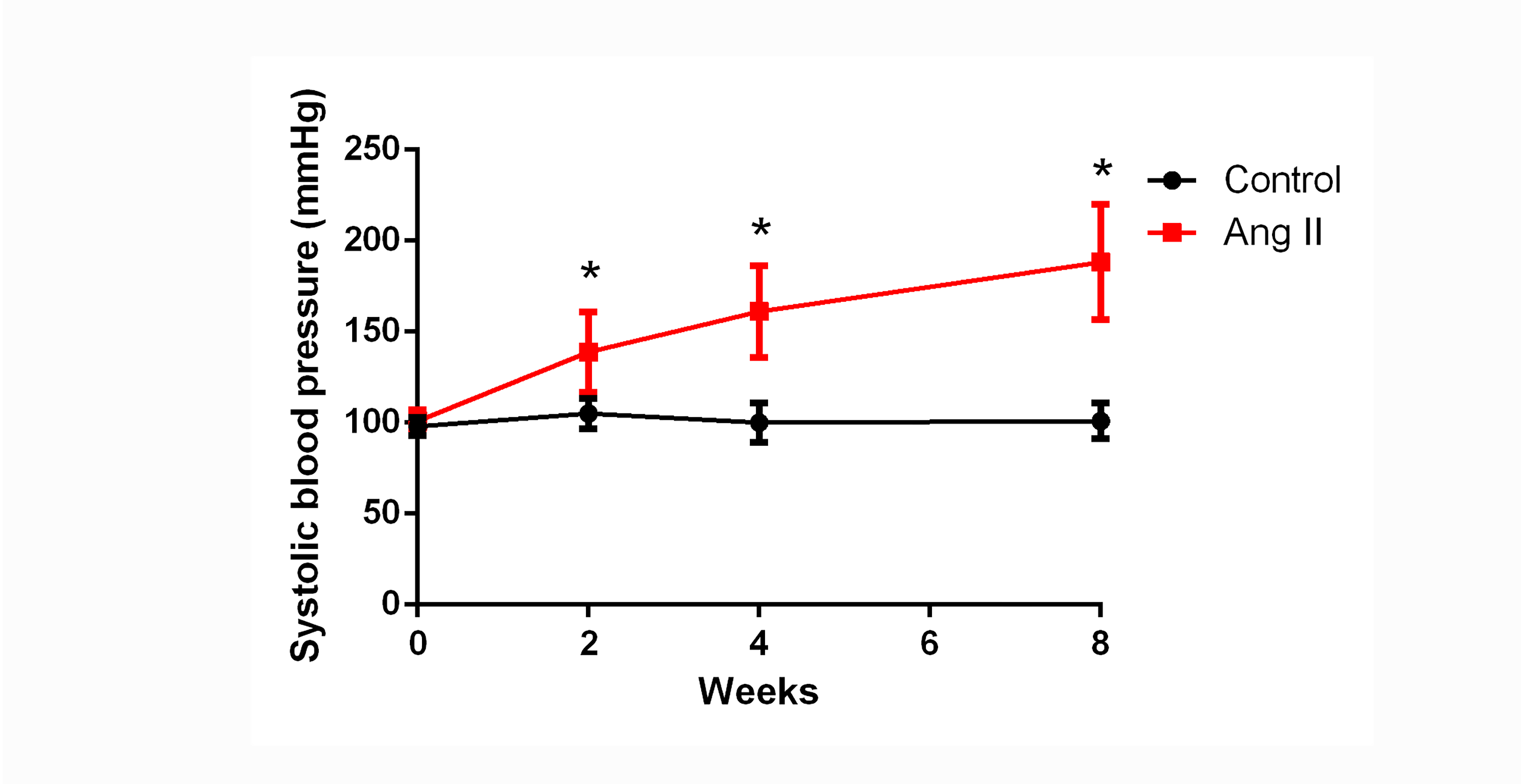

Supplement: Supplementary file 1 — Additional file 1. Changes in blood pressure in different groups of rats. [file 40360_2019_340_MOESM1_ESM.tif]
